# CDK5RAP2 Is an Essential Scaffolding Protein of the Corona of the *Dictyostelium* Centrosome

**DOI:** 10.3390/cells7040032

**Published:** 2018-04-23

**Authors:** Valentin Pitzen, Sophie Askarzada, Ralph Gräf, Irene Meyer

**Affiliations:** Department of Cell Biology, University of Potsdam, Karl-Liebknecht-Str. 24-25, 14476 Potsdam-Golm, Germany; valentin.pitzen@uni-potsdam.de (V.P.); askarzad@uni-potsdam.de (S.A.); rgraef@uni-potsdam.de (R.G.)

**Keywords:** centrosome, centriole, *Dictyostelium*, microtubules, mitosis

## Abstract

*Dictyostelium* centrosomes consist of a nucleus-associated cylindrical, three-layered core structure surrounded by a corona consisting of microtubule-nucleation complexes embedded in a scaffold of large coiled-coil proteins. One of them is the conserved CDK5RAP2 protein. Here we focus on the role of *Dictyostelium* CDK5RAP2 for maintenance of centrosome integrity, its interaction partners and its dynamic behavior during interphase and mitosis. GFP-CDK5RAP2 is present at the centrosome during the entire cell cycle except from a short period during prophase, correlating with the normal dissociation of the corona at this stage. RNAi depletion of CDK5RAP2 results in complete disorganization of centrosomes and microtubules suggesting that CDK5RAP2 is required for organization of the corona and its association to the core structure. This is in line with the observation that overexpressed GFP-CDK5RAP2 elicited supernumerary cytosolic MTOCs. The phenotype of CDK5RAP2 depletion was very reminiscent of that observed upon depletion of CP148, another scaffolding protein of the corona. BioID interaction assays revealed an interaction of CDK5RAP2 not only with the corona markers CP148, γ-tubulin, and CP248, but also with the core components Cep192, CP75, and CP91. Furthermore, protein localization studies in both depletion strains revealed that CP148 and CDK5RAP2 cooperate in corona organization.

## 1. Introduction

The centrosome is a non-membranous organelle organizing microtubule arrays in many eukaryotes. The evolutionary ancient centrosome type features a set of two centrioles consisting of a nine-fold symmetrical arrangement of short microtubules embedded in a pericentriolar matrix (PCM) consisting of scaffolding proteins that bind microtubule nucleation complexes. Due to their mode of duplication, there is one older so-called mother centriole, and a younger daughter centriole. During S-phase, each of them gives birth to one procentriole. Thus, centrioles duplicate once and only once per cell cycle. The PCM is organized by the mother centriole. Only after passage through mitosis where the two centrosomal entities separate to form the mitotic spindle, the daughter centriole becomes competent to organize a new centrosome on its own. This maturation process is called centriole–centrosome conversion and it depends on Cep152/Asl and polo-like kinase 1 [1,2]. Together with pericentrin (PCNT), Cep192/SPD-2, and CDK5RAP2/Cep215/Cnn the mature centriole organizes a radially arranged scaffold for the attachment of γ-tubulin-ring complexes (γ-TuRCs) [3]. Through these γ-TuRCs, the PCM nucleates the majority of microtubules. 

During G1 the mother centriole uses its distal appendages to dock at the plasma membrane, where it also functions as a basal body for the formation of the primary cilium [4]. The fact that organisms lacking cilia as locomotion organelles, such as many amoebozoans or fungi, contain no centrioles led to the hypothesis that the basal body-role of centrioles may have been their original function in the last eukaryotic common ancestor [5]. 

Within the eukaryotic supergroup of amoebozoans, centrosomes are best investigated in *Dictyostelium discoideum* amoebae [6]. As in vegetative animal cells, the centrosome is attached to the cytosolic face of the nucleus through a linkage structure involving the nuclear envelope protein Sun1 [7]. It consists of a cylindrical core structure composed of three layered disks, which is surrounded by a corona corresponding to the PCM of animal cells. The corona predominantly contains regularly spaced, electron-dense nodules at the base of 30–70 radially emanating microtubules [8,9]. The nodules contain γ-tubulin, and thus they harbor the nucleation complexes [10]. Among all known γ-tubulin ring complex-associated proteins (i.e., γ-tubulin, GCP2-6, GDP-WD/NEDD1 and MOZART1 [11]), only γ-tubulin, Spc97 (GCP2), and Spc98 (GCP3) could be identified in the *Dictyostelium* genome. Thus, *Dictyostelium* γ-Tubulin complexes (γ-TuCs) appear to be of a more simple composition than those of higher organisms. This is similar to the situation in budding yeast [12], however, with the difference that in *Dictyostelium,* purified soluble γ-TuCs are composed of γ-tubulin and Spc98 only, while Spc97 appears to join the two others only at the centrosome [13]. 

At the onset of mitosis, the layered core structure expands, while the corona disintegrates completely along with the attached microtubules [14]. Next, the core structure inserts into a fenestra opening in the nuclear envelope and the central layer disappears, while the formerly outer layers separate from each other within the nuclear envelope and represent the two mitotic spindle poles. The latter organize a central spindle under requirement of the microtubule crosslinker Ase1 [15]. Starting with the transition to anaphase, the plaque-like mitotic centrosomes fold back onto themselves, in a way that the microtubule-nucleating, formerly inner surfaces become oriented towards the outside. This process is completed in late telophase, when the central layer re-appears, the centrosomes are expelled from the nuclear envelope, and the microtubule-nucleating surface matures into a new corona. Yet γ-TuCs at this mitotic microtubule-nucleating surface have to be organized differently compared to the situation within the corona during interphase. This is based on the observation that the corona protein CP148 is not required to organize a central spindle, while it is essential to build up a new corona [16]. Thus, despite its role as a scaffolding protein within the corona, CP148 cannot be the only receptor for γ-TuCs. Recently, the identification of a CDK5RAP2 (=CDK5 regulatory subunit associated protein 2) orthologue in *Dictyostelium* by Sukumaran and co-workers brought up a candidate for a primary receptor for γ-TuCs that may interact with CP148 [17]. Mammalian CDK5RAP2, also called Cep215 or MCPH3, is one of the most ubiquitous centrosomal proteins with orthologues in insects (*Drosophila* centrosomin/cnn) and yeasts (Mto1p, Spc72p in *S. pombe* and *S. cerevisiae*, respectively). *Dictyostelium* CDK5RAP2 is encoded by *cepL* and was originally named Cep161, since it was recognized as a centrosomal binding partner of CP250/248 with an approximate molecular mass of 161 kDa [17]. To avoid confusion we prefer common names for orthologous proteins, so we use the well-introduced name CDK5RAP2 also for the *Dictyostelium* protein. *Dictyostelium* CDK5RAP2 contains a conserved centrosomin motif (CM1) for γ-TuC receptors [12,17,18]. Yet Sukumaran and co-workers focused on the effect of Cep161 overexpression on *Dictyostelium* development, where it interacts with the Hippo-related kinase SvkA, but did not further investigate its centrosomal functions, especially with regard to microtubule organization. 

In this work, we fill this gap of knowledge and show that CDK5RAP2 is required for the organization of the corona where it interacts not only with CP248, but also with CP148, γ-tubulin, and the core proteins Cep192, CP75, and CP91.

## 2. Materials and Methods

### 2.1. Vector Constructions and Expression of Recombinant Proteins for Immunization

The genomic sequence covering the complete coding sequence of CDK5RAP2 was amplified by PCR from genomic DNA using linker primers and cloned into the N-terminal GFP-fusion vector pIS77 [19] to yield pIS950 for G418 selection in *Dictyostelium*. In parallel this genomic fragment was cloned into the Flag-BirA*-fusion vector pIS936, which corresponds to pIS77 except that GFP is replaced by the Flag-tagged biotinylase BirA-R118G (=BirA*; [20]). To obtain the GFP-SPC97 fusion vector pIS1172 complete coding sequence ofSpc97 was also cloned into pIS77 using genomic DNA as a template for PCR.

For the CDK5RAP2-GFPki knockin strain we first created pIS1121, a general target vector for C-terminally GFP-tagged knockin strains. Thus, S65T-GFP and a 300 bp teminator sequence were cloned downstream of the first polylinker of pLPBLP [21], a plasmid which was originally designed for the generation of knock out strains by homologous recombination. In this vector, GFP is now followed by a floxed blasticidin cassette and a second polylinker. This design allows the simple generation of linear DNA-fragments, in which two arms of sequence homologous to a gene of interest are separated by a selection cassette. Here, a 600 bp fragment encoding the CDK5RAP2 C-terminus was amplified by PCR using linker primers and pIS950 as a template and cloned into pIS1121 using KpnI and EcoRI. In a second step 620 bp of 3′ untranslated sequence of the *cepL* gene were cloned into the second polylinker using PstI/BamHI. To create a linear fragment for the transformation into *Dictyostelium* cells (strain AX2), the final plasmid was digested with KpnI/BamHI. The obtained strain contains a gene replacement at the *cepL* locus resulting in *cepL* promoter-driven expression of CDK5RAP2-GFP in place of the endogenous untagged protein. The recombination vector lacks a promoter upstream of the CDK5RAP2-GFP coding sequence, so that only homologous integration into the *cepL* locus will give rise to the expression of full length GFP-fusion proteins. The resulting *Dictyostelium* strain CDK5RAP2-GFPki was also transformed with the CP148RNAi construct reported earlier [16].

In a similar way, the CP148-BioID2 knockin vector pIS1186 was built. First a general target vector, pIS1180, for C-terminally BioID2-tagged knockin strains was constructed. For this the coding sequence for the biotin ligase from *Aquifex aeolicus* was obtained by gene synthesis (Genart, Regensburg, Germany) and cloned into pLPBLP [21]. To guide the homologous recombination a 640 bp fragment corresponding to the CP148 C-terminus and 400 bp of 3′ untranslated sequence of the *cepG* gene were used.

Two different CDK5RAP2-RNAi constructs were prepared according to Martens et al. [22], where two arms of long inverted repeats are separated by a short stuffer fragment in between. The corresponding plasmids (pIS1109 and pIS1166) were constructed as described previously [23]. CDK5RAP2 sense fragments corresponding to base positions 1–478 (pIS1109) or 3427–4135 (pIS1166) of the coding sequence were amplified by PCR using SalI/BamHI linker primers and pIS950 as a template. In parallel the complementary reverse sequences were amplified using AflII/KpnI linker primers. Both PCR-fragments for each region were cloned into pIS193 yielding final vectors encoding an inverted CDK5RAP2 repeat with a short stuffer fragment originally derived from mCherry. The plasmids were transformed into a blasticidin-resistant *Dictyostelium* GFP-α-tubulin strain [24] and CDK5RAP2-RNAi clones were selected with G418. The RNAi constructs are expressed under control of the actin-6 promoter, which is rather weak, when cells are fed with bacteria during the selection process, but strong when clones are cultured in HL5c liquid medium. Transformation of both plasmids resulted in clones with identical phenotypes. Only data from cells with RNAi directed against the 3′-end of the coding sequence (pIS1166) are described in this work. 

For the C-terminal BioID fusion vector pIS1000, the coding sequence of BirA* was amplified by PCR from pIS936 [20] using BamHI/NsiI linker primers and cloned into p1ABr8A [25]. The coding sequence of TubC was taken from the GFP-TubC fusion vector [10] and transfered to pIS1000 using the KpnI/BamHI restriction sites.

For bacterial expression vectors, the sequence encoding the N-terminal 310 amino acids of CDK5RAP2 or amino acids 1262–1601 of Cep192 were amplified by PCR from genomic DNA using linker primers and cloned into pMalC2 (New England Biolabs, Frankfurt, Germany). For bacterial expression of MBP-CP55 (full length) previously described pMalC2-CP55 was used [26]. The fusion proteins were expressed at room temperature in *E. coli* Rosetta cells (NEB, Frankfurt, Germany) and purified by amylose agarose affinity chromatography according to the manufacturer’s manual (NEB, Frankfurt, Germany). They were then used for custom immunization of a rabbit (CDK5RAP2 and Cep192) or a rat (CP55) (Preclinics, Potsdam, Germany). For affinity purification of anti-CDK5RAP2, the MBP-fusion protein was coupled to NHS-activated Sepharose according to the manufacturer’s manual (GE Healthcare, Solingen, Germany).

### 2.2. Microscopy

Wide field fluorescence microscopy was performed essentially as described previously [16] using a Zeiss CellObserver HS system equipped with a PlanApo 1.4/100× objective, an Axiocam MRm Rev. 3 CCD camera and a piezo stage (Carl Zeiss Mikroskopie GmbH, Jena, Germany). Cells were fixed as indicated in the figure legends either for 2 min with methanol (at −20 °C) or 5 min with glutaraldehyde and stained according to Batsios et al. [27]. Glutaraldehyde is useful for GFP-fusion proteins and microtubules, while centrosomal core components usually require methanol, as stronger fixatives such as glutaraldehyde interfere with antigenicity. For deconvolution of image stacks from fixed cells, a point spread function (PSF) measured with 100 nm Tetraspeck beads (Thermo Fisher Scientific, Waltham, MA, USA) and the iterative algorithm of Axiovision 4.8 (Carl Zeiss Mikroskopie GmbH, Jena, Germany) were applied. For live cell imaging, cells were prepared as described previously [28] and viewed on a Zeiss CellObserver SD confocal spinning disk system with two Evolve EM-CCD cameras (Photometrics, Tucson, AZ, USA) and a LCI-PlanNeofluar 1.3/63× water immersion lens. The system is also equipped with a Rapp UGA-40-2L Galvo Scanner (Rapp Optoelectronics, Hamburg, Germany) employing its own 473 nm laser for photobleaching of user-defined regions of interest (FRAP). Data evaluation of FRAP experiments was performed according to Samereier et al. [28].

### 2.3. Other Methods

Cells were cultured in HL5c medium (Formedium, Hunsanton, UK). Sterile filtered glucose was added after autoclaving. Transformation of *Dictyostelium* amoebae by electroporation, SDS electrophoresis and Western blotting were carried out according to standard procedures [25,29]. Centrosomes or nuclei with attached centrosomes and were isolated as reported earlier [30]. BioID assays were performed as described recently [20,30].

### 2.4. Antibodies and Conjugates

Primary antibodies: anti-CDK5RAP2 (this work), anti-Cep192 (this work), anti-CP55 (rat, this work), anti-CP148 [16], anti-CP91 [31], anti-CP224 [32], YL1/2 [33], and anti-NE81 [34].

Secondary antibodies: all AlexaFluor conjugates were purchased from Thermo Fisher Scientific (Darmstadt, Germany), anti-mouse-Cy5 and anti-rat-Cy5 from Jackson Labs (Dianova, Hamburg, Germany), and enzyme conjugates for Western blotting from Sigma (Deisenhofen, Germany). Strept488 und AP.

## 3. Results

### 3.1. CDK5RAP2 Is a Component of the Centrosomal Corona

**Figure 1:**
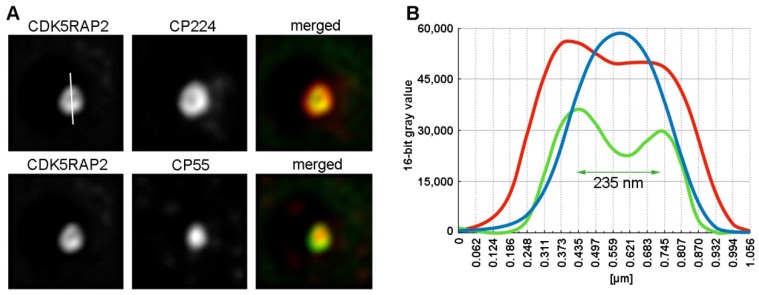
Subcentrosomal distribution of CDK5RAP2. Immunofluorescence deconvolution microscopy of AX2 control cells with rabbit anti-CDK5RAP2, rat anti-CP55 and mouse anti-CP224. Secondary antibodies against rabbit, rat and mouse antibodies were conjugated with AlexaFluor 568, AlexaFluor 488, and Cy5, respectively and cells were fixed with methanol. (**A**) The same representative centrosome is shown in all three channels as indicated. (**B**) Shows the corresponding intensity distributions for CDK5RAP2 (green), CP55 (blue) and CP224 (red) along the line shown in the upper left of (**A**).

Sukumaran et al. described CDK5RAP2 as a centrosomal component, however, its subcentrosomal localization and distribution in various cell cycle stages was not investigated. In our previous work, we could clearly show that resolution of wide-field deconvolution immunofluorescence microscopy is sufficient to address individual centrosomal components to either the corona or the layered core structure. In images of subdiffraction, 100-nm beads deconvolved with a measured PSF, measurement of the full-width-half-max of the intensity distribution of single beads indicated a resolution of ~170 nm with green fluorescence (Supplemental Figure S1). This technique enables the display of the corona as a fluorescent ring in an optical section through the center of the centrosome. As a molecular tool to investigate the distribution of endogenous CDK5RAP2 we raised a rabbit polyclonal antibody against the recombinant maltose-binding fusion protein expressed in *E. coli*. Image analysis of single optical sections of cells stained for the core marker CP55 and the corona marker CP224 clearly revealed a ring-like distribution of CDK5RAP2, similar to CP224 (Figure 1).

The diameter of the CP224 ring was slightly larger than that of the CDK5RAP2 ring, however, this may be due to the longer emission wavelength of the fluorescent dye coupled to the secondary antibody (Cy5 vs. AlexaFluor 568). As expected from its proposed involvement in microtubule organization, CDK5RAP2 is a component of the centrosomal corona. 

### 3.2. CDK5RAP2 Briefly Disappears from Centrosomes during Early Mitosis

**Figure 2:**
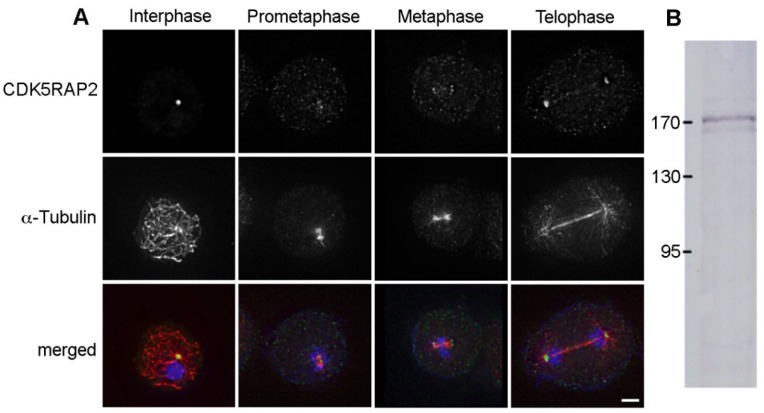
CDK5RAP2 is present at mitotic spindle poles. (**A**) Immunofluorescence microscopy of AX2 control cells in interphase and mitosis (as indicated) stained with rabbit anti-CDK5RAP2 and rat anti-α-tubulin. DNA staining with DAPI is shown in the merged image. Secondary antibodies were anti-rabbit AlexaFluor 488 and anti-rat AlexaFluor 568. Cells were fixed with glutaraldehyde. Maximum intensity projections of deconvolved images. To allow comparison of labeling intensities in mitotic vs. interphase cells, the maximum display intensity of the green channel was set using neighboring interphase cells. Bar = 2 µm. (**B**) Immunoblot of a nuclear extract of untransformed AX2 cells stained with anti-CDK5RAP2/anti-rabbit-alkaline phosphatase. Color detection was performed with nitroblue tetrazolium chloride (NBT) and bromo-chloro-indolyl-phosphate (BCIP).

Next, we studied CDK5RAP2 dynamics during mitosis. CDK5RAP2 was present also at mitotic spindle poles, however, labeling intensities in early mitosis up to metaphase were weaker than in interphase cells (Figure 2A). Anti-CDK5RAP2 also sometimes weakly stained the nucleus (as e.g., in Figure 6). Despite the presence of a predicted nuclear localization sequence in CDK5RAP2 at position 504–516 (predicted by http://nls-mapper.iab.keio.ac.jp), this weak nuclear staining likely reflects a cross-reaction of the purified antibody, as it did not change upon RNAi depletion of CDK5RAP2 (see below). In Western blots of isolated nuclei containing attached centrosomes, the antibody stained a major band at 170 kDa and more weakly, a second, slightly lower band, most probably representing a degradation product (Figure 2B). A very similar pattern was also reported by Sukumaran and co-workers using a different, mouse monoclonal antibody [17].

**Figure 3:**
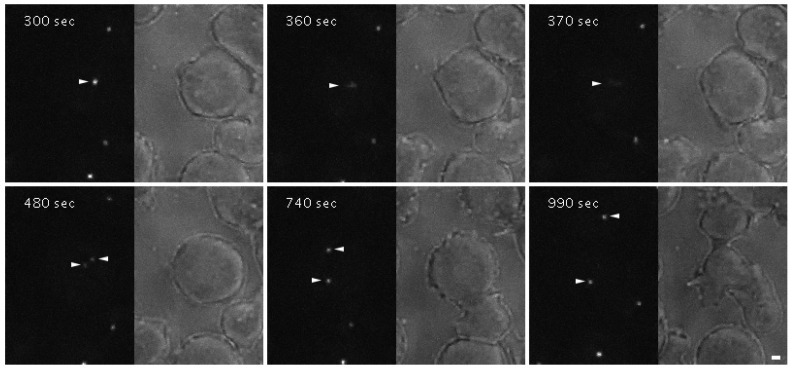
GFP-CDK5RAP2 briefly disappears from the centrosome during prophase. Confocal spinning disk live cell imaging of a mitotic GFP-CDK5RAP2 cell. Selected time points from Video S1 are displayed. Mitotic centrosomes are highlighted by arrowheads. Cells were viewed under agar overlay [35]. Bar = 2 µm.

To investigate this dynamic behavior more precisely in live cells, we expressed a GFP-CDK5RAP2 fusion protein. GFP-CDK5RAP2 behaved in a similar way to endogenous CDK5RAP2 (Supplementary Figure S2). Live cell imaging revealed that GFP-CDK5RAP2 almost disappears in prophase and early prometaphase, i.e., the stages when the corona disintegrates. It gradually re-appears from metaphase to telophase when microtubules are reorganized to form a central spindle, k-fibers, and astral microtubules (Figure 3, Video S1). 

### 3.3. CDK5RAP2 Exhibits Very Low Turnover at Interphase Centrosomes

**Figure 4:**
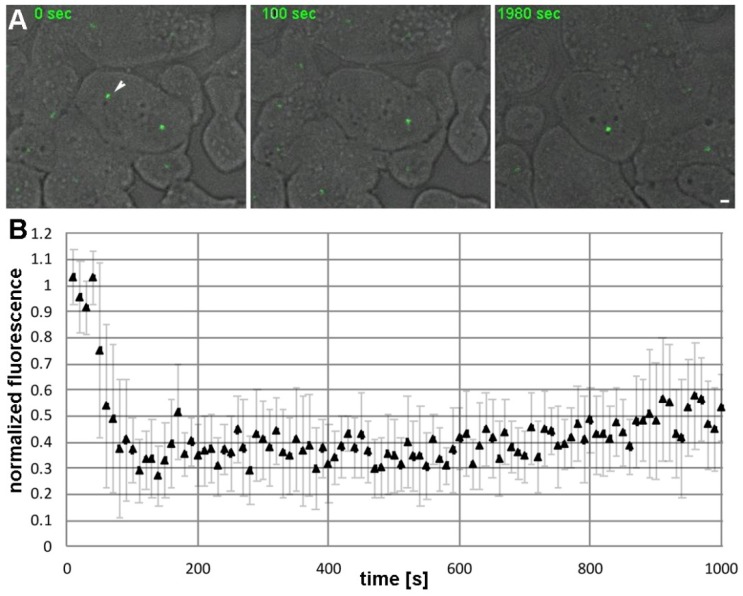
GFP-CDK5RAP2ki shows hardly any recovery after photobleaching. (**A**) Confocal spinning disk live cell imaging of GFP-CDK5RAP2ki cells. Selected time points from Video S2 are displayed. A maximum intensity projection of three confocal slices (z-distance 0.25 µm) is shown. The bleached centrosome is highlighted by an arrowhead. Cells were viewed under agar overlay [35]. Bar = 2 µm. (**B**) FRAP curve mean intensity values and error bars indicating SD (*n* = 10) are plotted against time.

This dynamic behavior of GFP-CDK5RAP2 in mitosis stands in contrast to its static behavior during interphase, which became apparent in FRAP analyses. First, experiments using our GFP-CDK5RAP2 strain revealed partial fluorescence recovery after photobleaching (not shown). However, since these cells still contain endogenous CDK5RAP2 and express GFP-CDK5RAP2 under control of a strong constitutively active promoter, this partial recovery may be a side effect of overexpression. To address this issue we created a knock-in strain, CDK5RAP2-GFPki, in which the endogenous CDK5RAP2 coding sequence was replaced by C-terminally GFP-tagged CDK5RAP2 expressed under control of the endogenous promoter. Using the knock-in strain, FRAP experiments showed virtually no fluorescence recovery after photobleaching (Figure 4, Video S2). This static behavior during interphase is in agreement with a role of CDK5RAP2 as a scaffolding protein for the organization of microtubule-nucleation complexes, in analogy to the role of CDK5RAP2 as a PCM-organizing scaffolding protein in mammalian centrosomes.

### 3.4. RNAi Depletion of CDK5RAP2 Disrupts Corona Organization and Radial Microtubule Arrays

**Figure 5:**
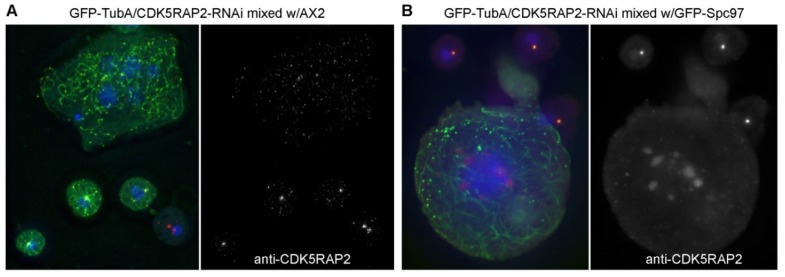
CDK5RAP2RNAi cells contain display disrupted microtubule arrays and enlarged nuclei. Immunofluorescence microscopy of GFP-TubA/CDK5RAP2RNAi cells mixed with AX2 control cells (**A**) or GFP-Spc97 cells (**B**) stained with rabbit anti-CDK5RAP2. DNA-staining with DAPI is shown in the merged image. The secondary antibody was anti-rabbit-AlexaFluor568. Cells were fixed with glutaraldehyde. Maximum intensity projections of deconvolved images are displayed. Bar = 2 µm.

A proposed function for CDK5RAP2 is expected to be essential for viability, so it is not surprising that several attempts to knock out *cepL* failed, as it holds true for most other centrosomal components in *Dictyostelium* as well. Thus, we depleted CDK5RAP2 by RNAi, essentially using the strategy of Martens and co-workers, in which a plasmid encoding inverted repeat yields dsRNA, which is then cleaved by dicer to generate siRNAs [22]. Two different RNAi constructs were made, one directed against the 5′ end of the coding sequence and a further one directed against its 3′-end (see Section 2.1). The resulting RNAi strains were indistinguishable and only data referring to the second variant are shown. We expected effects on microtubule organization, so we employed our well-established GFP-α-tubulin strain as the target strain for RNAi to yield CDK5RAP2-RNAi cells. Using the actin-6 promoter to drive expression of dsRNA, we were able to induce expression by switching culture conditions, from growth on bacterial lawns to axenic liquid culture. After induction, CDK5RAP2-RNAi cultures grew poorly and even stopped growing after several days. To check for effectiveness of CDK5RAP2 depletion, cells were mixed with either an equal number of AX2 control cells, or GFP-Spc98 control cells with green centrosomes [13] and stained with anti-CDK5RAP2. In fluorescence microscopy, RNAi cells were readily distinguishable from control cells by their green-fluorescent microtubules (Figure 5). The extent of CDK5RAP2 depletion in fluorescence microscopy varied strongly from cell to cell, ranging from no considerable CDK5RAP2 depletion to its complete absence from remaining centrosomal foci. 

**Figure 6:**
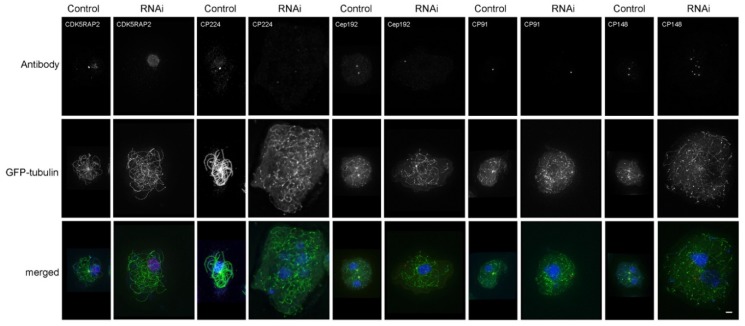
CDK5RAP2RNAi causes centrosome disruption. Immunofluorescence microscopy of GFP-TubA/CDK5RAP2RNAi cells stained with the indicated antibodies, in comparison to GFP-α-tubulin control cells. DNA staining with DAPI is shown in the merged images. Secondary anti-rabbit/rat/mouse antibodies were all AlexaFluor568. Cells were fixed with methanol. Maximum intensity projections of deconvolved images are displayed. Bar = 2 µm.

The latter cells also showed disorganized microtubules (25%, *n* = 133) and often multiple, enlarged nuclei, indicating increased ploidy. This phenotype was very reminiscent of that observed upon CP148-RNAi [16]. Next, we tested whether CDK5RAP2 RNAi affected the presence of other centrosomal marker proteins of the core and corona structure, namely CP91, Cep192, CP148, and CP224 (Figure 6).These analyses clearly revealed a disintegration of centrosomes in RNAi cells, along with disturbed microtubules. Centrosomal corona proteins such as CP224 and CP148 were either dispersed, or concentrated at several cytoplasmic foci. 

Core proteins such as CP91 and Cep192 were usually present at one single spot per nucleus, however, not in tight association with the nucleus itself. These results indicate that loss of CDK5RAP2 causes disintegration of the corona, and dissociation of the remaining core structure from the nucleus, while dispersed microtubule-nucleation complexes are still capable of nucleating microtubules.

### 3.5. GFP-CDK5RAP2 Overexpression Elicits Supernumerary Cytosolic MTOCs

**Figure 7:**
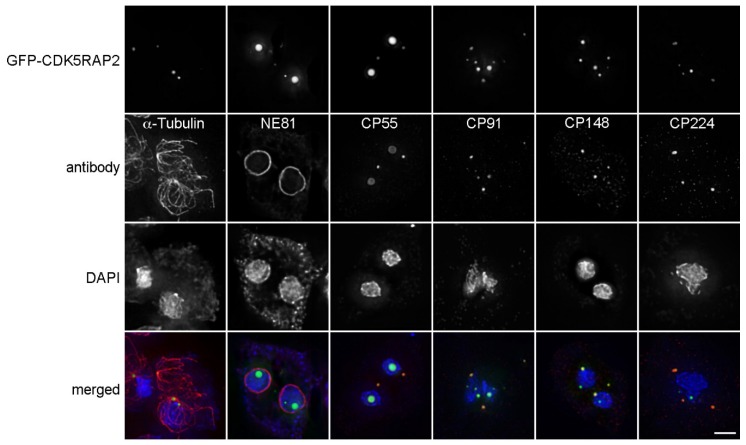
Overexpressed GFP-CDK5RAP2 is concentrated in discrete foci in the cytosol or nucleus. Immunofluorescence microscopy of GFP-CDK5RAP2 cells stained with the indicated antibodies. DNA was stained with DAPI. Secondary anti-rabbit/rat/mouse antibodies were conjugated with AlexaFluor 568. Cells were fixed with methanol. Maximum intensity projections of deconvolved images are displayed, except for the GFP-CDK5RAP2 sample co-stained for NE81 (lamin, nuclear envelope marker, [34]), where only the optical section through the center of the nucleus is shown, to show that there are indeed GFP-CDK5RAP2 foci inside the nucleus. Bar = 2 µm.

Not only depletion, but also overexpression of CDK5RAP2 caused informative aberrations. In contrast to the knock-in-strain (CDK5RAP2-GFPki) which was used for FRAP experiments, the first mentioned GFP-CDK5RAP2 cells express the fusion protein under control of the strong actin-6 promoter, and often showed fluorescent cytosolic and nuclear aggregates (Figure 7). 

The presence of nuclear GFP-CDK5RAP2 aggregates indicates the functionality of the predicted NLS sequence (see above). Nuclear GFP-CDK5RAP2 foci were generally not associated with corona markers such as CP148, CP224, and microtubules, but rather with varying amounts of the core markers CP91 and CP55. In contrast, cytosolic GFP-CDK5RAP2 aggregates in addition contained corona markers, and were capable of serving as microtubule organizing centers. This indicates that overexpressed GFP-CDK5RAP2 is able to recruit not only centrosomal core components, but also microtubule-nucleation complexes. 

### 3.6. BioID Analysis Reveals Centrosomal Interactors of CDK5RAP2

**Figure 8:**
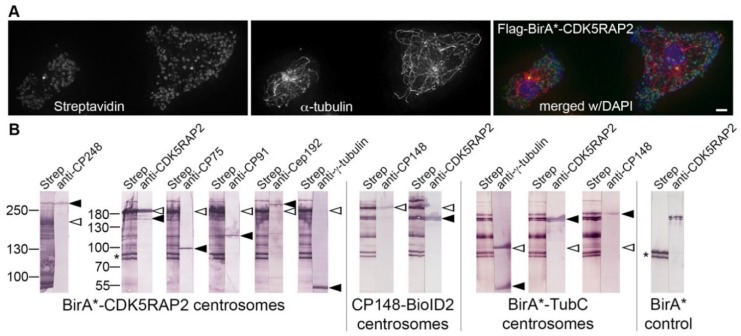
Centrosomal interactions of biotinylase-tagged CDK5RAP2 in the BioID assay. (**A**) Immunofluorescence microscopy ofFlag-BirA-tagged CDK5RAP2RNAi cells stained with anti-α-tubulin and streptavidin-AlexaFluor 488. DNA staining with DAPI is shown in the merged image. The secondary antibody was anti-rat AlexaFluor 568. Cells were fixed with glutaraldehyde. Maximum intensity projections of deconvolved images are displayed. Bar = 2 µm. (**B**) BioID Western blot analysis according to [30]. Centrosome extracts prepared from the cell lines indicated at the bottom were electrophoresed and blotted. Single lanes were cut from broad blots, stained individually with streptavidin-alkaline phosphatase (strep) or antibodies as indicated on top, and re-aligned after staining as shown. Bands were visualized using conjugates with alkaline phosphatase and NBT/BCIP color detection. Positions of the respective fusion proteins always showing self-biotinylation are marked by open arrowheads. Positions of endogenous, biotinylated proteins are labeled by filled arrowheads. The position of the double band representing biotinylated mitochondrial proteins is highlighted by an asterisk.

The occurrence of centrosome-like structures upon overexpression of GFP-CDK5RAP2 suggests a high capability of binding other centrosomal proteins and subcomplexes. Since many centrosomal proteins are expressed at low levels, are virtually absent from cytosolic extracts, and interact only at the centrosome, protein interaction studies by co-IP assays are limited. Thus, we pursued our successful method to identify centrosomal interactors by proximity-dependent biotin identification (BioID) [20,30]. In this method, the protein of interest is fused to a promiscuous biotin ligase which biotinylates any lysine residue within a proximity of ~10 nm [36]. Thus, due to this very short distance, biotinylated substrate proteins most likely are direct binding partners of the tagged protein of interest. Two different biotinylases were used: First, BioID* derived from *E. coli* [37]; and second, the smaller BioID2 derived from *Aquifex aeolicus* [38]. For the purpose of this study, we created three different strains: (1) a FLAG BirA*-CDK5RAP2 strain expressing the N-terminally tagged protein driven by the actin-6 promoter; (2) a CP148-BioID2 knock-in strain expressing the C-terminally tagged fusion protein under control of the endogenous promoter; and (3) BirA*-TubC expressing N-terminally tagged γ-tubulin driven by the actin-6 promoter. In fluorescence microscopy, all fusion proteins localized to centrosomes when stained with fluorescently labeled streptavidin (representatively shown for Flag-BirA*CDK5RAP2 in Figure 8A). Due to the presence of the endogenous mitochondrial, biotinylated carboxylase MccA [20], streptavidin stained also mitochondria. *Dictyostelium* cells express three endogenously biotinylated proteins, that are also visible in Western blots of strains expressing BirA* only, i.e., methylcrotonyl-CoA carboxylase alpha (MccA) (77 kDa), acetyl-CoA carboxylase (AccA) (257 kDa), and propionyl-CoA carboxylase alpha (PccA) (80 kDa) (www.dictybase.org, [39]) (Figure 8B, BirA*control). Aside from these endogenous proteins (only two of them—MccA and PccA—are visible in our Western blots), centrosome preparations of the control strain showed no further biotinylated proteins, underlining the high specificity of the BioID method. We have identified binding partners of CDK5RAP2 by parallel immunoblotting of isolated centrosome extracts with specific antibodies directed against selected centrosomal proteins, and a streptavidin-alkaline phosphatase conjugate (Figure 8B). Proteins biotinylated by Flag-BirA*-CDK5RAP2 included CP75, CP91, Cep192, and—rather weakly—γ-tubulin. Since CP148 has a similar molecular weight as Flag-BirA*-CDK5RAP2 and hence the band will be masked, biotinylation of CP148 could not be shown using this strain. Therefore, we chose the reciprocal approach and used our CP148-BioID2 strain to demonstrate biotinylation of CDK5RAP2, and thus the interaction of these two corona organizers. The low degree of biotinylation of γ-tubulin in the Flag-BirA*-CDK5RAP2 strain was unexpected, since CDK5RAP2 binds γTuRCs in other species, plus the *Dictyostelium* protein contains a predicted γ-tubulin complex interaction domain. Thus, we employed the BirA*-TubC strain to reveal CDK5RAP2 biotinylation by biotinylase-tagged γ-tubulin. Western blot analysis clearly demonstrated strong biotinylation of CDK5RAP2 and CP148. Surprisingly the fusion protein itself, which commonly displays the strongest biotinylation signal, was biotinylated rather weakly and endogenous γ-tubulin was not biotinylated at all. This behavior strongly suggests that γ-tubulin is a poor substrate for biotinylation in this assay, possibly due to a lack of accessible lysine residues. This could also explain the virtual absence of γ-tubulin biotinylation in the Flag-BirA*-CDK5RAP2 strain. 

### 3.7. CDK5RAP2 and CP148 Are Scaffolding Proteins Cooperating in Corona Organization

**Figure 9:**
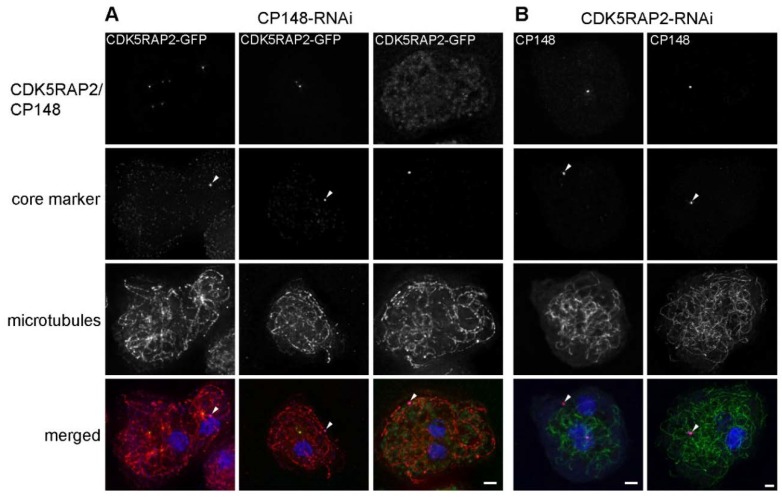
CDK5RAP2RNAi and CP148RNAi cause similar centrosome disruptions. Immunofluorescence microscopy of CDK5RAP2-GFP/CP148RNAi (**A**) and GFP-TubA/CDK5RAP2RNAi (**B**) cells stained with the indicated antibodies. DNA-staining with DAPI is shown in the merged images. In (**A**) primary and secondary antibodies were anti-α-tubulin/anti-rat-Atto647n and anti-Cep192 (core marker; arrowhead)/anti-rabbit-AlexaFluor 568, in (**B**) anti-CP55 (core marker; arrowhead)/anti-rat-AlexaFluor 568 and anti-CP148/anti-rabbit-Alexa 647. Cells were fixed with methanol. Maximum intensity projections of deconvolved images are displayed. Bars = 2 µm.

The strong interaction of CDK5RAP2 and CP148 in the BioID assay and the similarity of the phenotypes upon depletion of the respective proteins by RNAi, both indicate a cooperation of both proteins in centrosome and microtubule organization. To study the mutual dependence of both proteins with regard to their association with centrosomal core structures, we studied the effect of CP148 depletion on CDK5RAP2 localization in the CDK5RAP2-GFP knock-in strain (Figure 9). 

As upon CDK5RAP2-RNAi, core structures in these CP148-RNAi cells were often located at a far distance from the nucleus in the cytoplasm. In cells displaying a clear microtubule disruption phenotype, these core structures were usually not associated with CDK5RAP2. Occasionally, cells contained cytosolic MTOCs associated with radial microtubule arrangements and CDK5RAP2, but devoid of core markers, which were instead found at unrelated locations, without being associated with radial microtubule arrangements. Vice versa, in CDK5RAP2-RNAi cells we observed core structures with and without CP148. However, neither was associated with radial microtubule arrangements. This indicates that both proteins are required for the organization of the corona around the core structure, and also for attachment of the whole centrosome to the nucleus.

## 4. Discussion

In addition to *Dictyostelium* CDK5RAP2, several corona components—i.e., γ-tubulin, Spc97, Spc98, CP224, TACC, EB1, CP148, and CP248—have been characterized on a molecular and functional level [10,13,16,19,23,24,25]. While the role of CP248 (also known as CP250 and NAB350; [19,40,41]) still remains elusive, the γ-TuC components γ-tubulin, Spc97, and Spc98; and the microtubule regulators CP224 (XMAP215-family), TACC, and EB1 are involved in microtubule nucleation and polymerization, respectively, and CP148 is required for the assembly of the centrosomal corona. With regard to the latter, our results clearly demonstrate that it interacts with CDK5RAP2 in this function. Here CDK5RAP2 is required as a scaffolding protein for γ-TuCs and for corona assembly in a similar manner as it is known for CDK5RAP2 in the pericentriolar matrix of animal cells. Thus, the *cepL* gene product, initially named Cep161, behaved like a *bona fide* orthologue of the CDK5RAP2-family of centrosomal proteins, hence our naming. Our functional characterization of CDK5RAP2 and its interactions now allows to draw a clearer picture of corona structure and biogenesis and its evolutionary relationship to the pericentriolar matrix. 

### 4.1. CDK5RAP2 and CP148 in Corona Organization and Their Relationship to PCM Proteins

Since the corona is the functional equivalent of the pericentriolar matrix, it is reasonable that the process of corona formation around the core layers involves a similar set of proteins as in centriole–centrosome conversion in animal cells [2]. Four proteins are of prime importance in forming a scaffold for γ-TuRCs, i.e., Cep152/Asl, pericentrin, Cep192, and CDK5RAP2 [3]. Two of them, Cep152 and pericentrin, could not be identified clearly in *Dictyostelium.* Due to a low degree of sequence conservation, and to the dominance of coiled coil motifs in many centrosomal proteins, centrosomal orthologues between evolutionary distant species are difficult to identify. With regard to its size, CP148 was a possible candidate for a Cep152 orthologue. This idea is also supported by the fact that, within the *Dictyostelium* proteome, it is most similar to Cep152/Asl. However, considerable amino acid similarity was found only within a 140 aa sequence stretch of the central coiled-coil region (48% similarity, 23% identity as indicated by BLAST). A further similarity consists of a predicted calmodulin-binding IQ-domain at the end of the first third of primary sequence of both proteins. Yet, functional IQ-domains are also present within the pericentrin homology group, e.g., in yeast pericentrin (Spc110p; [42]). Currently, we cannot assess whether CP148 is orthologous to pericentrin or to Cep152, or whether it combines the functions of both in one protein. Budding yeast, for example, employs two different γ-TuC receptors on each side of the spindle pole body. While at the inner, nuclear plaque this function is performed by the pericentrin-like Spc110p, the CDK5RAP2-like Spc72p anchors γ-TuCs at the outer, cytosolic plaque. From this point of view, the existence of only one γ-TuC receptor in *Dictyostelium* is not surprising, as all centrosomal microtubule nucleation sites face the cytosol during interphase. 

With regard to the organization of γ-TuCs, *Dictyostelium* and budding yeast are more similar to each other than to all other model organisms. No orthologues of GCP4, 5, and 6 could be identified, which are present even in fission yeast, and required to build the large γ-tubulin ring complex (γ-TuRC) of higher eukaryotic cells. Budding yeast employs the small γ-TuSC complexes composed of only γ-tubulin, GCP2, and GCP3 to nucleate microtubules [42], and in *Dictyostelium* all soluble complexes are made up of γ-tubulin and GCP3 (Spc98) only. The third component, GCP2 (Spc97), appears to join the others exclusively at the centrosome.

### 4.2. Mutual Dependence of CDK5RAP2 and CP148 in Corona Organization

Our studies in *Dictyostelium* have demonstrated that the organization and integrity of the centrosomal corona depends both on CDK5RAP2 and CP148. This is based on the similarity of the respective protein depletion phenotypes, which are characterized by disintegration of the corona and randomly arranged microtubules. Yet, the actual presence of microtubules shows that neither of the two proteins is required for microtubule nucleation and growth, but rather for the recruitment of nucleation complexes to one location, i.e., the centrosome. 

The fact that efficient biotinylation of target proteins in the BioID assay requires a proximity of less than 10 nm and the strong biotinylation of CDK5RAP2 by CP148-BioID2, strongly suggests a direct interaction between both partners. Yet, this interaction must be regulated in early mitosis, since CP148, like CP248, disappears from mitotic spindle poles [16,40], while CDK5RAP2 disappears only briefly during prometaphase, corresponding to the short time period between disintegration of the corona and initiation of spindle formation. Assuming that CP148 is an orthologue of Cep152, the only polo-like kinase in *Dictyostelium*, Plk, is the most likely regulator [2]. Here, it remains to be shown whether the two Plk-phosphorylation sites predicted in the CP148 primary sequence are functional in vivo. 

Live cell imaging studies in many mitotic CP148-RNAi cells have clearly shown that CP148 is not required for mitotic spindle formation [16]. In contrast, despite numerous trials we never managed to find CDK5RAP2-RNAi cells exhibiting a strong microtubule disarrangement phenotype, that were still capable of entering mitosis, suggesting that CDK5RAP2 is required for mitotic entry and spindle formation. This could also explain why the growth of CDK5RAP2-RNAi cell cultures tends to stagnate once expression of the double-stranded RNA construct is induced, and why we barely found mitotic cells in fixed specimens. The idea of a general requirement of CDK5RAP2 to anchor microtubule nucleation complexes is also supported by the observation that some cytosolic GFP-CDK5RAP2-foci observed upon overexpression as well as some cytosolic CDK5RAP2-GFP-foci observed upon CP148-RNAi treatment were capable of organizing radial microtubule arrangements. Such arrangements were never observed upon CDK5RAP2-RNAi, or at cytosolic foci of overexpressed GFP-CP148 [16]. In other words, at low CDK5RAP2 levels CP148 alone is not capable of organizing such microtubule arrangements, not even when associated with core structures, while lack of CP148 does not prevent formation of core-free cytosolic MTOCs containing CDK5RAP2. 

### 4.3. Putative Role of the CDK5RAP2 Interactor CP248 within the Corona

Among the known corona components, the role of CP248 is most elusive, as its knockout has no obvious effect on microtubule organization [40]. Yet, the interaction of CP248 with CDK5RAP2 (this work and [17]) indicates a role in corona organization as well. With regard to its size, its overall predicted protein structure and its absence from mitotic spindle poles all are reminiscent of C-Nap1. The idea that CP248 may represent the orthologue of C-Nap1 is also supported by its cross-reaction with antibodies against human C-Nap1 [43]. In mammalian cells, C-Nap1 localizes to proximal centriole ends and is required for centriole cohesion, in cooperation with CDK5RAP2 [44]. At the G2/M transition, phosphorylation of C-Nap1 (together with other proteins) by the NIMA-related kinase Nek2 allows separation of the two centrosomes to form two opposing spindle poles [44,45,46]. In analogy, CP248 phosphorylation by *Dictyostelium* Nek2 may be a prerequisite for disintegration of the corona, and has to precede the separation of the two outer core layers later forming the two opposing spindle poles in *Dictyostelium*. CP248 contains several predicted Nek2 phosphorylation motifs (ELM; [47]), and its disappearance and reappearance at mitotic centrosomes is correlated with the disintegration and re-formation of the corona. Future phosphorylation experiments with recombinant Nek2 [48] will be conducted to address this question.

Knockout of CP248 also affected the distribution of Sun1, which was less focused at the pericentrosomal region [40]. As all other organisms containing centrosomes [7,49], Sun1 plays a crucial role in centrosome-nucleus attachment. Yet, its interactors at the centrosome are still elusive, since no true KASH-domain proteins could be identified so far. Although effects on centrosome-nucleus attachment were not reported in CP248 null cells [40], the altered Sun1 distribution in CP248 null cells indicates a role of the corona in centrosome–nucleus attachment during interphase. This is further supported by the observation that depletion of both CP148 [16] and CDK5RAP2 (this work) lead to the detachment of remaining core structures from nuclei.

### 4.4. Emergence of Cytosolic MTOCs upon GFP-CDK5RAP2 Overexpression

High expression levels of GFP-CDK5RAP2 caused the emergence of cytosolic MTOCs in addition to the nucleus-associated centrosome. In the first view it could be possible that this effect is not only caused by a too-high concentration of the fusion protein but also by some functional impairment due to the bulky GFP-tag. However, the centrosome-associated centrosome that carries the fusion protein as well appears and functions normally in these cells. Thus we assume that supernumerary MTOC are solely due to excess protein amounts. In *Dictyostelium*, supernumerary MTOCs can be elicited by either overexpression or depletion of various centrosomal proteins. As long as they do not attach to the nuclear envelope and remain cytosolic, they usually do not affect mitotic progression or cell viability. The semi-closed mitosis in *Dictyostelium* precludes microtubule connections between kinetochores and cytosolic MTOCs. In some instances—e.g., in case of overexpression of CP224, CP91, CP39, NdrC, and Nek2 [30,31,48,50,51] or lack of CP91 and CP75 [30,31]—these cytosolic MTOCs appear like *bona fide* centrosomes from a structural point of view, i.e., they are composed of a corona organized around a core structure, and contain all common centrosomal marker proteins. Yet, CP55-null cells are an example for cytosolic MTOCs clearly lacking a core structure [26]. In case of CDK5RAP2, cytosolic GFP-CDK5RAP2 foci organize radial microtubule arrays, and contain the centrosomal core markers CP55 and CP91, albeit to varying extents. Although these variations may indicate that cytosolic MTOCs do not represent bona fide centrosomes, it became clear that CDK5RAP2 has an inherent capacity of organizing not only the corona but also whole centrosomes. Yet, a final conclusion whether these MTOCs are true centrosomes has to await ultrastructural analysis.

## Supplementary Materials

The following are available online http://www.mdpi.com/2073-4409/7/4/32/s1. Figure S1: Resolving power of widefield deconvolution microscopy, Figure S2: GFP-CDK5RAP2 is present at mitotic spindle poles, Video S1: GFP-CDK5RAP2 briefly disappears from the centrosome during prophase, Video S2: GFP-CDK5RAP2ki shows hardly any recovery after photobleaching.
