# Supplementary figures and images for: CDK5RAP2 Is an Essential Scaffolding Protein of the Corona of the Dictyostelium Centrosome
Source: Cells. 2018 Apr 23;7(4):32. doi: 10.3390/cells7040032 (PMC5946109; doi:10.3390/cells7040032)

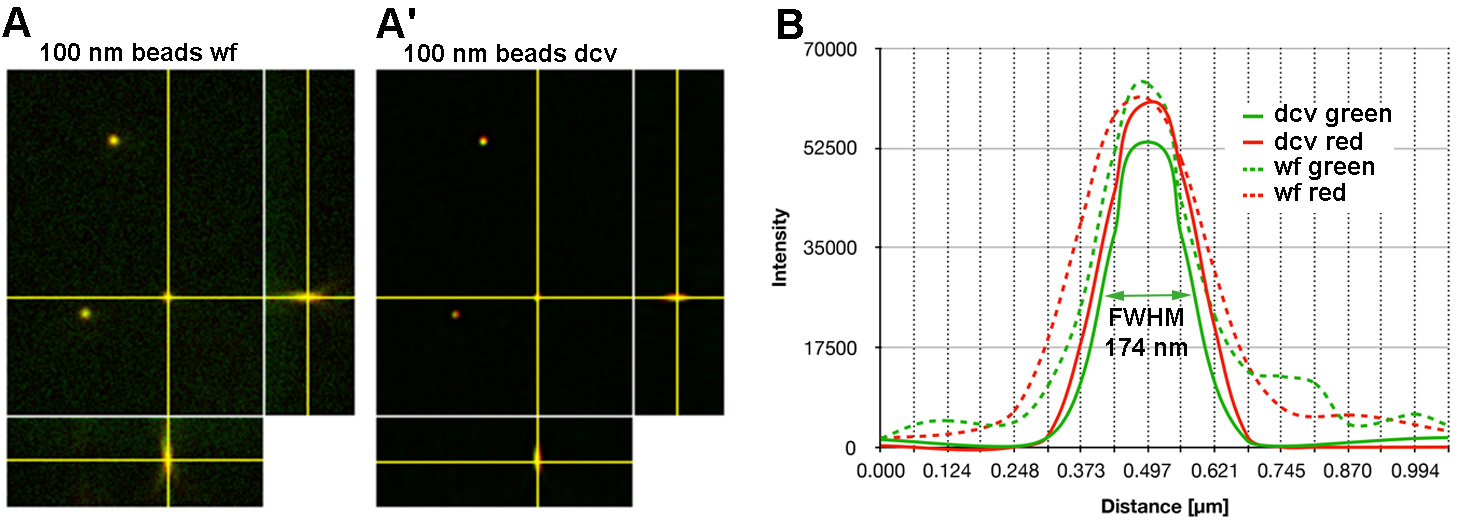

Supplement: Supplementary file 1 [file cells-07-00032-s001.zip › FigS1.jpg]

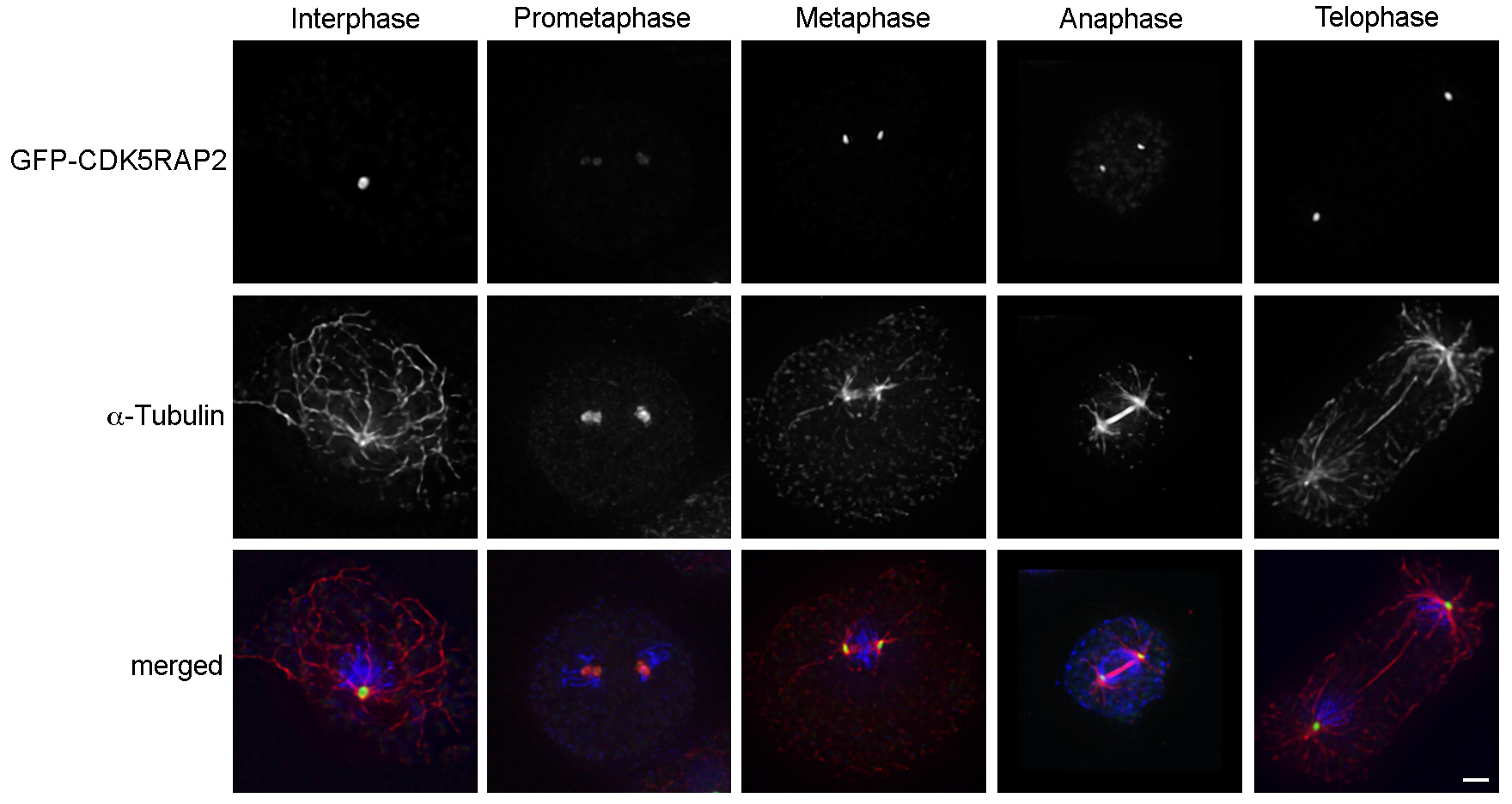

Supplement: Supplementary file 1 [file cells-07-00032-s001.zip › FigS2-GFP-Cep161-MT-rot-Montage.jpg]
